# Rapid Microbiological Assessment in Raw Milk: Validation of a Rapid Alternative Method for the Assessment of Microbiological Quality in Raw Milk

**DOI:** 10.3390/foods9091186

**Published:** 2020-08-27

**Authors:** Nicla Marri, Francesca Losito, Loris Le Boffe, Gilberto Giangolini, Simonetta Amatiste, Lorenza Murgia, Alyexandra Arienzo, Giovanni Antonini

**Affiliations:** 1 Istituto Zooprofilattico Sperimentale del Lazio e della Toscana *M. Aleandri* (IZSLT), 00178 Rome, Italy; nicla.marri@izslt.it (N.M.); gilberto.giangolini@izslt.it (G.G.); simonetta.amatiste@izslt.it (S.A.); 2 Interuniversity Consortium Istituto Nazionale Biostrutture e Biosistemi (INBB), 00136 Rome, Italy; losito.francesca00@gmail.com (F.L.); loris.leboffe@uniroma3.it (L.L.B.); 3 Science Department, Università degli Studi Roma Tre, 00146 Rome, Italy; lorenza.murgia@uniroma3.it (L.M.); alyexandraarienzo@gmail.com (A.A.)

**Keywords:** raw milk, microbiological safety, microbiological quality, food safety, dairy

## Abstract

The consumption of dairy products and the dairy industry are one of the main global agri-food sectors for its size, economic importance, and level of technology. Microbiological quality of pasteurized milk or other milk products is dependent on microbiological quality of raw milk. A variety of microbiological count methods is available for monitoring the hygienic quality of raw milk. Among them, the pour plate method is the official essay for counting the number of colony-forming units in milk samples according to International Organization for Standardization (ISO) No. 4833-1:2013. The aim of the present study is the validation of the Micro Biological Survey (MBS) method, against the reference plate-count method, for the assessment of the microbiological quality of raw milk. This comparative study, performed in collaboration with the Istituto Zooprofilattico Sperimentale del Lazio e della Toscana M. *Aleandri* (IZSLT), demonstrates the accuracy of this alternative method for the determination of total viable bacterial count in cow’s raw milk. The results obtained with the MBS method highlight its potential as a valid tool for reliable microbiological analysis in dairy industries.

## 1. Introduction

Milk has always been considered an essential food for its nutritional value, especially for children and adolescents, and a significant portion of human diet is nowadays based on the consumption of dairy products, making the dairy industry one of the main global agri-food sectors for its size, economic importance, and level of technology [1].

Microbial contamination can generally occur from various sources during the milking procedure—from dairy farms to the milk and cheese industry—therefore, it is essential to monitor microbiological characteristics along the whole production chain to prompt preventive actions to protect human health [2]. High microbial counts in raw milk are responsible for quality defects in pasteurized milk, UHT processed milk, dried skim milk, butter, and cheese [3].

Current regulations define “raw milk” as milk not receiving thermal treatment above 40 °C or any equivalent process [4].

The raw milk may be used for producing heat-treated milk, cheese, or milk-based products, or it can be directly sold from self-service vending machines. Although the Italian Health Ministry with Decree 12 December 2012 ordered that vending machines should bear the notice ‘‘Milk must be consumed after boiling’’, some consumers may ignore this advice [5,6]. The European Community Regulation (EC) No 178/2002, laying down the general principles and requirements of food law, establishing the European Food Safety Authority and laying down procedures in matters of food safety, sets rules, procedures, and a pioneering approach regarding food safety [7]. The Regulation (EC) No 853/2004, currently in force, requests routine evaluation of raw milk’s microbiological quality through the assessment of the mesophilic flora upon plate culture and defines the microbiological criteria for raw milk as: ≤100,000 colony-forming units CFU/mL upon plate count at 30 °C for raw cow’s milk, ≤1,500,000 CFU/mL for other dairy species and ≤500,000 CFU/mL when the final destination of milk from other species does not include heat treatment [4].

A variety of microbiological count methods is available for monitoring the hygienic quality of raw milk. Among them, the pour plate method is the official essay for counting the number of CFUs in milk samples according to International Organization for Standardization (ISO) No. 4833-1:2013 [8]. The flow cytometry technique is the most common method used for evaluating the hygienic quality of raw milk, which estimates the total number of bacteria present in milk according to ISO 21187:2004 [9].

New methods for the detection and enumeration of microorganisms have been developed to provide accurate, rapid tools to flank standard plate culture protocols. In this context, the application of Micro Biological Survey (MBS) method should be considered as an additional safety measure, which cannot replace reference methods. The MBS method, developed by MBS Srl and Roma Tre University (Rome, Italy), is a colorimetric, culture-based system that allows us to perform quantitative and qualitative microbiological analyses in the absence of either dedicated facilities or specialized personnel. The MBS method measures the catalytic activity of the redox enzymes in the main metabolic pathways of bacteria through a redox indicator that changes color according to the oxidative state of the medium. The time required for the color change is inversely related to the logarithm of bacterial concentration, and this allows us to obtain an unequivocal correlation between the observed enzymatic activity and the number of viable cells present in the samples. Selectivity is assured by the peculiar composition of each reagent and from the temperature of analysis: only target bacteria can grow in the reaction vials. Analyses are performed into ready-to-use, sterile, disposable vials, designed for in situ use, following a simple and straightforward protocol in order to reduce human error associated with sample manipulation [10,11].

The aim of the present study is the validation of the MBS method, against the reference plate-count method, for the assessment of the microbiological quality of raw milk on naturally contaminated samples. All validation experiments were carried out in partnership with Istituto Zooprofilattico Sperimentale del Lazio e della Toscana M. *Aleandri* (IZSLT), which provided raw milk samples and logistic resources, and in collaboration with MBS Srl, which provided the MBS analytical kit.

## 2. Materials and Methods

Total viable mesophilic bacteria were evaluated in 173 samples of cow’s raw milk following the reference method, according to ISO 4833-1: 2013, and the MBS method, in parallel. All samples for both methods were analyzed in duplicate.

According to the reference method, for each raw milk sample, 1 mL of the undiluted sample and selected serial decimal dilutions were plated on skim milk agar using the pour plate method; plates were then incubated at 30 °C for 72 h. Only plates displaying results falling within the 10–324 colonies were considered for the count according to ISO 7218:2013, then results underwent verification by G^2^ factor test for the proportionality of the counts [12].

Concomitantly, the MBS method was performed using the total viable count (TVC) vials for the quantification of total viable mesophilic bacteria. All vials were produced by MBS Srl (Rome, Italy). According to the proprietary user’s manual, TVC vials were inoculated with 1 mL of the undiluted raw milk samples. After inoculation, vials were incubated for up to 30 h at 30 °C in the MBS multireader, a thermostatic optical reader that automatically detects the color change of the MBS vials and calculates the bacterial concentration in the analyzed sample. The absence of color change after 30 h indicates the absence of the microorganisms of interest. MBS results were expressed as time (h).

## 3. Results

Results obtained using the reference method were grouped into four classes according to the detected bacterial concentration, arranged in ascending order: ≤100,000 CFU/mL (102 samples, 57.3%), 101,000–500,000 CFU/mL (43 samples, 26.5%), 501,000–1,000,000 CFU/mL (13 samples, 8.1%), and >1,000,000 CFU/mL (15 samples, 8.1%). The lowest recorded value was 2500 CFU/mL, while the highest was 4,700,000 CFU/mL. Results were then converted into log_10_. The statistical analysis was performed using MedCalc for Windows (MedCalc 12, MedCalc Software Ltd., Ostend, Belgium). The repeatability of the MBS method was verified on two levels (first level mean: 0.80 log_10_; second level mean 8.62 log_10_), performing 10 replicates for each level. The repeatability’s standard deviation was Sr = 0.03 log_10_ and Sr = 0.02 log_10_ for the first and second levels, respectively.

**Figure 1:**
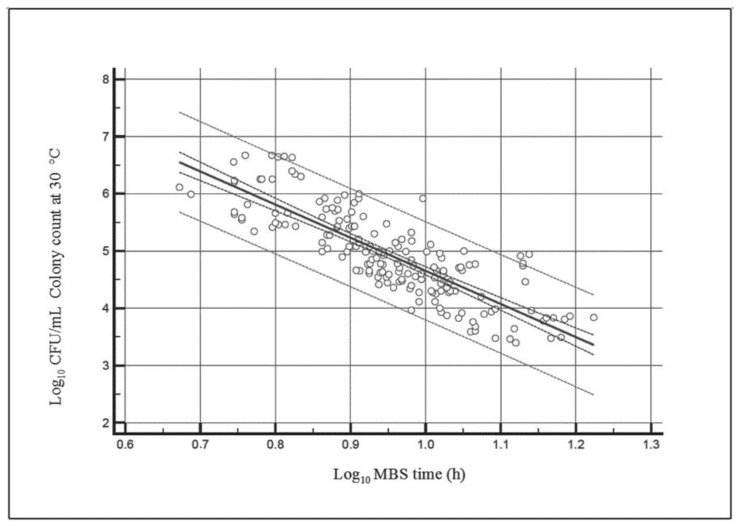
Regression line (R^2^ = 0.69) obtained plotting the decimal logarithms of the bacterial concentration values yielded with the reference method (Log CFU/mL) and the decimal logarithm of the time required for Micro Biological Survey (MBS) vials color change yielded with the MBS method (Log h).

The average values for the colony count at 30 °C obtained with the plate-count reference method was compared with the average values obtained with the MBS method for each of the 173 samples of raw cow’s milk analyzed. The regression line obtained plotting the log of bacterial concentration values obtained with the reference method, and the log of results obtained with the MBS method (time required for the MBS vials to change color), shown in Figure 1, confirms the linearity of the MBS method and provides a correlation line defined by the equation:

(1) $$\frac{\log_{10}\mathrm{CFU}}{\mathrm{mL}}=-5.7889\;\times {\;\log}_{10}\mathrm{MBStime}+10.4419$$

This equation, allowing us to calculate the number of bacteria present into a sample on the basis of the time required for color change, can be stored into the MBS multireader software, allowing it to automatically calculate the number of bacteria present in a sample from the time required for color change.

It is worth noting that the analytical time required for the change of color with the MBS method in raw milk samples ranges from 5 h (for highly contaminated samples, i.e., >10^6^ CFU/mL) up to 15 h (for less contaminated samples, i.e., <10^4^ CFU/mL)—see Figure 1. This analytical time should be compared with the 72 h required for looking at the number of colonies present in skim milk agar plates according to ISO 7218:2013. Thus, the MBS method resulted from about 5 up to 15-fold faster than the classical method.

Table 1 summarizes regression main parameters.

**Figure 2:**
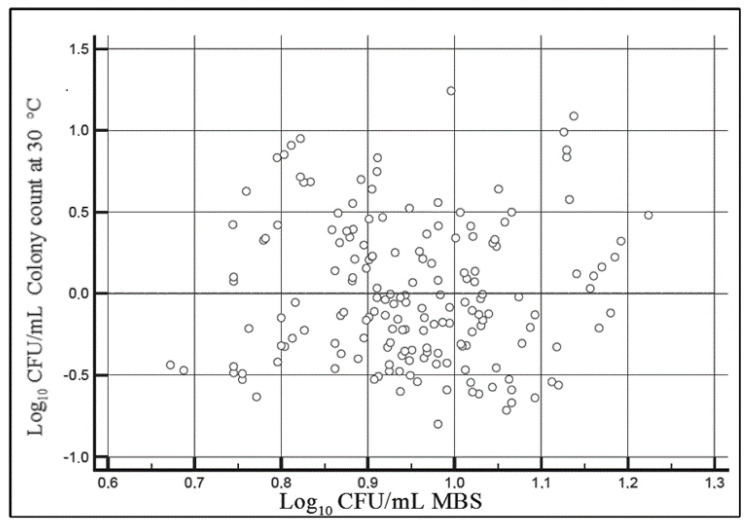
Determination of residues of linear regression obtained comparing results yielded with the two methods. CFU: colony-forming units.

Although the number of tested samples may appear to not be very high, the Pearson value (r) obtained in the study is equal to 0.83, being intermediate between the values 0.80 and 0.85 with minimum samples 217 and 149, respectively, according to UNI EN ISO 21187:2004 [9]. The relatively low R^2^ value depends on intrinsic variability of both the classical method and MBS method together with small differences between tested samples. After that, the determination of the residuals of the previously calculated linear regression was carried out, demonstrating that the distribution of residues obtained through the use of the above-mentioned equation displays a normal pattern (Figure 2). The results obtained with the two methods were also graphically compared by the Bland–Altman test. The confidence interval (CI) at 95% of the mean difference was identified using the following equation:

(2) CI=Mean ± 1.96 × s

Mean = mean of differences of reference and alternative method. s = standard deviation of differences.

**Figure 3:**
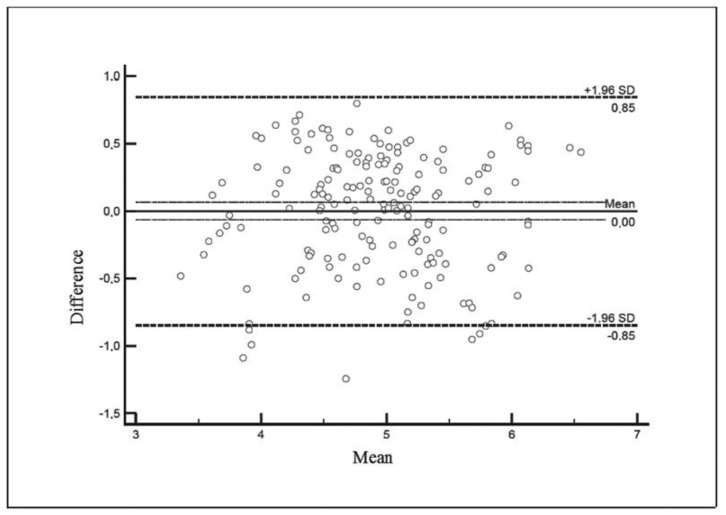
Bland–Altman test. The mean values of the two methods are shown on the X axis. The difference of the two values is shown on the Y axis.

The upper and lower limits were defined as CI = ±0.85. The results obtained with the Bland–Altman test (Figure 3) show a substantial reliability with respect to the interchangeability of the two methods.

**Figure 4:**
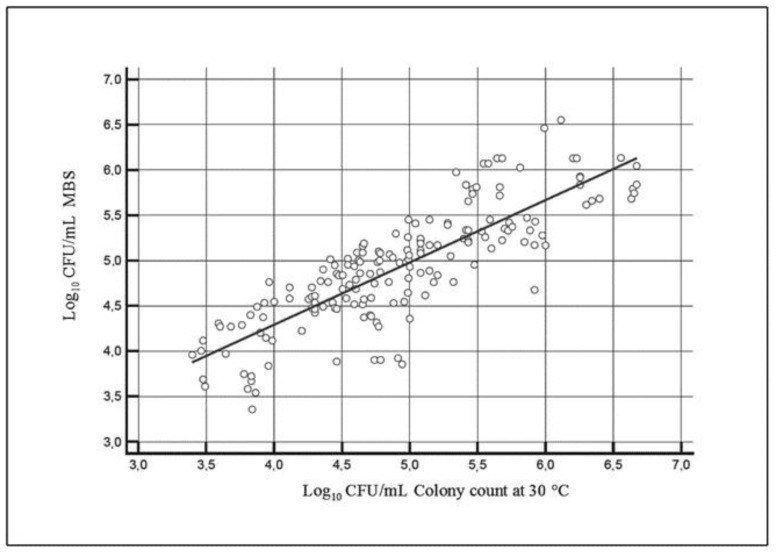
Relation between the results (log CFU/mL) of the alternative MBS method and the reference method for total bacterial count at 30 °C in bovine raw milk.

Reference results were plotted against alternative method results and converted to the same unit of measurement of reference method (Figure 4). The MBS method’s accuracy was evaluated according to ISO 16297:2020, calculating 95% CI for the difference observed between results obtained with the two methods within 6 categories identified by a 0.5 log_10_ interval. The CI acceptance limit, as specified by ISO 16297:2020, displays values far from the required CI ± 0.8 log_10_ (1.05 log_10_ CFU/mL and −1.37 log_10_ CFU/mL, respectively) [13].

Table 2 shows CI results for each category. Results obtained in the 8.10 × 10^3^–776 × 10^3^ CFU/mL range comply with the required CI; CIs from the 2.5 × 10^3^–8.0 × 10^3^ CFU/mL range and the 832 × 10^3^–4.677 × 10^3^ CFU/mL range display higher values (1.05 log_10_ CFU/mL and −1.37 log_10_ CFU/mL, respectively) that do not comply with the required CI. This suggests an overestimation of results obtained with the MBS method for the first group results, compared to the reference method, and an underestimation for the last group. However, the threshold value (100,000 CFU/mL) for colony count at 30 °C specified by regulations (Regulation (EC) No 853/2004) falls within the 8.10 × 10^3^–776 × 10^3^ CFU/mL range, which displays satisfactory CI [4].

## 4. Discussion

This comparative study performed between the MBS method and the reference method demonstrates the accuracy of the alternative method for the determination of total viable bacterial count in raw cow’s milk. These data, although preliminarily limited to raw milk, highlight the potential of the MBS method as a valid tool for reliable microbiological analysis for the dairy industries and for producers of non-pasteurized milk and cheeses. Its features, including simplicity (just put raw milk to the vial, no need of sample preparation, no need of expert technicians nor of a microbiological laboratory [14]), accuracy (as accurate as classical methods [15,16]), and rapidity (5 to 15-fold faster than classical methods, as here reported), make it particularly suitable for use in small and medium-sized dairy companies for routine analysis and to improve the quality of their products [17].

## 5. Conclusions

The application of the MBS method should be considered as an additional tool to increase safety and quality of dairy products, thought should not replace officially approved analytical methods, which must be used in accordance with current legislation.

## Figures and Tables

**Table 1:** Main parameters defining the regression line obtained plotting reference method and Micro Biological Survey (MBS) results.

N Samples	Pearson (r)	R^2^	Sy:x	Intercept (Standard Error)	Slope (Standard Error)
173	0.83	0.69	0.43	0.29	0.30

**Table 2:** Confidence interval (CI) results for the 6 categories identified in the study.

Group Range (log_10_ CFU/mL)	3.40–3.90	3.92–4.40	4.41–4.90	4.91–5.40	5.41–5.89	5.92–6.67
Group range (CFU/mL × 1000)	2.5–8.0	8.1–25	26–80	81–251	257–776	832–4.677
N samples	19	22	47	34	30	21
CI upper	1.05	0.77	0.77	0.59	0.57	0.23
CI lower	−0.37	−0.04	−0.54	−0.78	−0.77	−1.37
